# Association between interleukin-22 genetic polymorphisms and bladder cancer risk

**DOI:** 10.6061/clinics/2015(10)05

**Published:** 2015-10

**Authors:** Tao Zhao, XiaoHou Wu, JiaJi Liu

**Affiliations:** I Chongqing Medical University, YongChuan Hospital, Department of Urology, YongChuan, Chongqing, China; II The First Affiliated Hospital of Chongqing, Medical University, Department of Urology, Chongqing, China

**Keywords:** Interleukin-22, Gene Polymorphism, Bladder Cancer, Case-Control Study

## Abstract

**OBJECTIVE:**

The cytokine interleukin-22 (IL-22), which is produced by T cells and natural killer cells, is associated with tumorigenesis and tumor progression in cancers. However, the role of IL-22 in bladder cancer has not been investigated.

**MATERIALS AND METHODS:**

A prospective hospital-based case-control study comprising 210 patients with pathologically proven bladder cancer and 210 age- and gender-matched healthy controls was conducted. The genotypes of 3 common polymorphisms (-429 C/T, +1046 T/A and +1995 A/C) of the *IL-22* gene were determined with fluorogenic 5' exonuclease assays.

**RESULTS:**

Patients with bladder cancer had a significantly higher frequency of the *IL-22* -429 TT genotype [odds ratio (OR)=2.04, 95% confidence interval (CI)=1.19, 3.49; *p*=0.009] and -429 T allele (OR=1.42, 95% CI=1.08, 1.87; *p*=0.01) than the healthy controls. These findings were still significant after a Bonferroni correction. When stratifying according to the stage of bladder cancer, we found that patients with superficial bladder cancer had a significantly lower frequency of the *IL-22* -429 TT genotype (OR=0.48, 95% CI=0.23, 0.98; *p*=0.04). When stratifying according to the grade and histological type of bladder cancer, we found no statistical association. The *IL-22* +1046 T/A and *IL-22* +1995 A/C gene polymorphisms were not associated with the risk of bladder cancer.

**CONCLUSION:**

To the authors' knowledge, this is the first report documenting that the *IL-22* -429 C/T gene polymorphism is associated with bladder cancer risk. Additional studies are required to confirm this finding.

## INTRODUCTION

Bladder cancer is the most common cancer of the urinary tract and is the fourth most commonly diagnosed malignancy in men in the United States. It is estimated that 74,000 new cases of bladder cancer are expected to occur in the United States in 2015 1. Smoking tobacco and occupational exposure to chemical carcinogens have been established as the strongest risk factors for developing bladder cancer 2-4. It is now commonly accepted that the cause of bladder cancer is the multi-factorial interaction of environmental triggers with genetic susceptibility 5-9. Genome-wide association studies have identified multiple susceptibility loci associated with bladder cancer risk 10-13.

Interleukin-22 (IL-22) is a member of the *IL-10* family or *IL-10* superfamily, a class of potent mediators of cellular inflammatory responses 14. IL-22 is synthesized by different cell types, including T- and natural killer (NK)-cells and has been reported to mediate crosstalk between inflammatory cells and keratinocytes 15-17. IL-22 is also associated with tumorigenesis and tumor progression in cancers 18,19. The human *IL-22* gene is located on the long arm of chromosome 12, on 12q15, approximately 52 and 99 kbp upstream from the *IL-26* and interferon loci, respectively and has the same transcriptional orientation as these two adjoining genes 15. Several single nucleotide polymorphisms (SNPs) have previously been identified in the *IL-22* gene 20-27.

However, the role of IL-22 in bladder cancer has not been investigated. The aim of this study was to investigate the association between *IL-22* gene polymorphisms (-429 C/T, +1046 T/A and +1995 A/C) and the risk of bladder cancer in a Chinese population.

## MATERIALS AND METHODS

### Study population

A prospective hospital-based case-control study of 210 patients with pathologically proven bladder cancer and 210 age- and gender-matched healthy controls was conducted in the Department of Urology of the YongChuan Hospital of ChongQing Medical University. The healthy control subjects were randomly selected when they attended a clinic for a routine examination. All the bladder cancer cases were staged according to the TNM staging system of the Union Internationale Contre le Cancer. Bladder tumors were graded using the World Health Organization (WHO) classification. All the individuals were interviewed by trained nurse-interviewers using a structured questionnaire that asked for information regarding the patients' gender, age, smoking status and occupational and other exposure histories. All parts of the study were approved by the Institutional Ethical Committee of the ChongQing Medical University and informed consent according to the Declaration of Helsinki was obtained from all the participants or their families/surrogates.

### DNA extraction and genotyping

DNA was extracted from peripheral blood lymphocytes using a commercially available Qiagen kit (Qiagen Inc., Valencia, CA, USA). The genotypes of 3 common polymorphisms (-429 C/T, +1046 T/A and +1995 A/C) of the *IL-22* gene were determined with fluorogenic 5' exonuclease assays (TaqMan, Applied Biosystems, Foster City, CA, USA). The primer and probe sequences for the 5'-exonuclease assays of the *IL-22* polymorphisms are listed in Table 1. The polymerase chain reaction (PCR) was performed in a Primus 96 plus thermal cycler using a total volume of 5 µl containing 2.5 µl of Universal-MasterMix, 0.125 µl of 40x Assay-by-Design mix, 0.375 µl of H_2_O and 2 µl of DNA. The reactions were overlaid with 15 µl of mineral oil. The cycling parameters were as follows: 10 min at 94°C for primary denaturation, followed by 40 cycles of 20 s at 92°C and 1 min at 60°C. Fluorescence was measured in a Lambda Fluoro 320 Plus plate reader (MWG Biotech AG, Germany).

### Statistical analysis

The data were presented as the means±standard deviation (SD) or as percentages for categorical variables. Differences between continuous variables were assessed using Student's *t* test, while those between categorical variables were evaluated using Pearson's *x*^2^ test. A multivariate logistic regression analysis was used to calculate crude and adjusted odds ratios (OR) and 95% confidence intervals (CI) for the association between each *IL-22* polymorphism and bladder cancer risk. Statistical significance was set at a nominal *p*-value <0.05 for all comparisons. SAS version 9.1 (SAS Institute, Cary, NC) was used for all the statistical tests.

## RESULTS

The characteristics of the bladder cancer cases and healthy control subjects are presented in Table 2. A univariate analysis was performed and the results indicated that smoking (*p*=0.01) was associated with bladder cancer (Table 2). No significant difference was found between the bladder cancer cases and healthy controls regarding sex or age (Table 2). Regarding the tumor stage of the cases, 134 (63.8%) cases were invasive (T2-T4) bladder cancer and 76 (36.2%) were superficial (Tis-T1) bladder cancer. Regarding the tumor grades of the cases, 65 (31.0%) cases were low grade (G1) bladder cancer and 145 (69.0%) cases were high grade (G2+G3) bladder cancer. The analysis of the histological types of the cases found that 53 (25.2%) cases were nonpapillary bladder cancer and 157 (74.8%) were papillary bladder cancer.

The genotype frequencies were in agreement with the Hardy-Weinberg equilibrium. The patients with bladder cancer had a significantly higher frequency of the *IL-22* -429 TT genotype (OR=2.04, 95% CI=1.19, 3.49; *p*=0.009) and -429 T allele (OR=1.42, 95% CI=1.08, 1.87; *p*=0.01) than the healthy control subjects (Table 3). The findings remained significant after a Bonferroni correction was implemented. When stratifying according to the stage of bladder cancer, we found that superficial bladder cancer had a significantly lower frequency of the *IL-22* -429 TT genotype (OR=0.48, 95% CI=0.23, 0.98; *p*=0.04) (Table 4). When stratifying according to the grade and histological type of bladder cancer, we found no statistical association (Table 4). The *IL-22* +1046 T/A and *IL-22* +1995 A/C gene polymorphisms were not associated with the risk of bladder cancer (Table 3).

## DISCUSSION

In this study, we investigated the association between three common polymorphisms (-429 C/T, +1046 T/A and +1995 A/C) of the *IL-22* gene and the risk of bladder cancer in a Chinese population. This prospective hospital-based case-control study revealed that the *IL-22* -429 C/T gene polymorphism is associated with bladder cancer risk. To the best of our knowledge, this is the first report in the literature that evaluated the association between *IL-22* gene polymorphisms and the risk of bladder cancer.

There is accumulating evidence that genetics plays a key role in the susceptibility to and clinicopathologic characteristics of bladder cancer. Nine meta-analyses, each of which analyzed between four and twenty-four studies, have provided evidence that the following polymorphisms are associated with increased bladder cancer risk: *NQO1* Pro187Ser; *PSCA* rs2294008 (C>T); *XRCC1* Arg399Gln (especially in non-Asian populations); *MMP-2* -1306 C/T; *MMP-9* -1562 C/T; *XPD* Lys751Gln; *ERCC2* Arg156Arg; Asp312Asn; Lys751Gln; *CCND1* G870A (which may modulate the risk of bladder cancer in conjunction with tobacco smoking); *XPC* A499V (in Caucasian populations); *CYP1A1* polymorphisms (especially the 11599G>C, 2455A>G, 3810T>C, and 113T>C polymorphisms in Asians); *MDM2* SNP309T>G (among Caucasians) 28-36.

The mechanisms of action for the *IL-22* -429 TT genotype and T allele as risk factors for bladder cancer are still unclear. IL-22 may play a role in controlling tumor growth and tumor progression by inhibiting signaling pathways that promote tumor cell proliferation, such as ERK1/2 and AKT phosphorylation 37. The *IL-22* -429 C/T gene polymorphism has been associated with the risks for various cancers. Genetic polymorphisms and plasma levels of *IL-22* contribute to the development of non-small cell lung cancer 38. Recently, a case-control study found that the *IL-22* -429 C/T gene polymorphism might be associated with the risk and multifocality of papillary thyroid cancer 26. In 561 colon cancer cases and 722 population controls, an association study suggested that the rs1179251 SNP in IL-22 was associated with the risk of colon cancer 23.

Several limitations to this study should be mentioned. First, these results should be interpreted with caution because the study subjects were Chinese; therefore, the study does not permit extrapolation of the results to different ethnic populations. Second, this is a hospital-based case-control study. Therefore, a selection bias could not be avoided and the subjects may not be representative of the general population. Third, the sample size of our study was relatively small and may not have had adequate statistical power in detecting significant differences. Finally, we did not evaluate the relationships of the 3 common polymorphisms (-429 C/T, +1046 T/A and +1995 A/C) to the plasma levels of IL-22, which may potentially reflect the disease state of patients. Therefore, the association of the IL-22 polymorphisms with plasma levels of IL-22 should be further investigated.

In conclusion, to the authors' knowledge, this is the first report documenting that the *IL-22* -429 C/T gene polymorphism is associated with bladder cancer risk. Additional studies are required to confirm this finding.

## Figures and Tables

**Table 1:** Primer and probe sequences for the 5'-exonuclease assays of *IL-22* polymorphisms.

SNPs	rs2227485	rs1182844	rs1179246
Exchange	-429 C/T	+1046 T/A	+1995 A/C
Forward Primer	AAAATGAGTCCGTGACCAAAATGC	CCACCTATGAGACTTCCCTATCAGT	GAAAAAGCCTTCCTGCCTAATGG
Reverse Primer	ACACAATTGTTTTGTCTTAGTAGAGTTCAGAT	CACTAAAGGAAAAGGAAAGCTGTGTTT	GGTGCTGCCTAAAGGTCAGA
Wild-type Probe	FAM-CTCCTATAGTGACTGAGTAA-NFQ	VIC-AAACTTACTAGTAGGTATGACTC-NFQ	VIC-TGAACAGAGTTATCTGCCTC-NFQ
Mutant Probe	VIC-CTCCTATAGTGGCTGAGTAA-NFQ	FAM-CTTACTAGTAGGAATGACTC-NFQ	FAM-AACAGAGTTAGCTGCCTC-NFQ

SNPs: single nucleotide polymorphisms.

**Table 2:** Distribution of the characteristics of bladder cancer cases and healthy control subjects.

	Cases (n=210)	Controls (n=210)	*p*
Sex (Male/Female)	134/76	127/83	0.48
Age (Years)	61.3±9.8	60.7±9.4	0.52
Smoking (Ever/Never)	111/99	85/125	0.01
Tumor stage (%)			
Invasive (T2-T4)	134(63.8)		
Superficial (Tis-T1)	76(36.2)		
Tumor grade (%)			
High (G2+G3)	145(69.0)		
Low (G1)	65(31.0)		
Tumor histological type (%)			
Papillary	157(74.8)		
Nonpapillary	53(25.2)		

**Table 3:** Genotype and allele frequencies of *IL-22* gene polymorphisms among bladder cancer cases and healthy controls.

Genotypes	Cases (n=210)	Controls (n=210)	OR (95% CI)	*p*
-429 CC	68(32.4)	79(37.6)	1.00(Reference)	
-429 CT	84(40.0)	98(46.7)	1.00(0.64,1.54)	0.99
-429 TT	58(27.6)	33(15.7)	2.04(1.19,3.49)	0.009
-429 C allele frequency	220(52.4)	256(60.9)	1.00(Reference)	
-429 T allele frequency	200(47.6)	164(39.1)	1.42(1.08,1.87)	0.01
+1046 TT	90(42.8)	97(46.2)	1.00(Reference)	
+1046 TA	77(36.7)	74(35.2)	1.12(0.73,1.72)	0.60
+1046 AA	43(20.5)	39(18.6)	1.19(0.71,2.00)	0.52
+1046 T allele frequency	257(61.2)	268(63.8)	1.00(Reference)	
+1046 A allele frequency	163(38.8)	152(36.2)	1.12(0.85,1.48)	0.43
+1995 AA	68(32.4)	65(30.9)	1.00(Reference)	
+1995 AC	101(48.1)	98(46.7)	0.99(0.64,1.53)	0.95
+1995 CC	41(19.5)	47(22.4)	0.83(0.49,1.43)	0.51
+1995 A allele frequency	237(56.4)	228(54.3)	1.00(Reference)	
+1995 C allele frequency	183(43.6)	192(45.7)	0.92(0.70,1.20)	0.53

OR: odds ratio; CI: confidence interval.

**Table 4:** Stratification analysis of the *IL-22* -429 C/T polymorphism in bladder cancer cases.

		CC		
		Cases (n=210)	n (%)	OR (95% CI)	*p*
Tumor stage	210	68(32.4)	1(Reference)	
Invasive	134	40(29.9)	0.92(0.59,1.44)	0.72
Superficial	76	28(36.8)	1.14(0.68,1.90)	0.62
Tumor grade	210	68(32.4)	1(Reference)	
High	145	48(33.1)	1.02(0.67,1.57)	0.92
Low	65	20(30.8)	0.95(0.54,1.68)	0.86
Tumor histological type	210	68(32.4)	1(Reference)	
Papillary	157	49(31.2)	0.96(0.63,1.47)	0.86
Nonpapillary	53	19(35.8)	1.11(0.61,2.00)	0.74

		CT		
		Cases (n=210)	n (%)	OR (95% CI)	*P*
Tumor stage	210	84(40.0)	1(Reference)	
Invasive	134	46(34.3)	0.86(0.56,1.31)	0.48
Superficial	76	38(50.0)	1.25(0.79,1.99)	0.35
Tumor grade	210	84(40.0)	1(Reference)	
High	145	55(37.9)	0.95(0.64,1.42)	0.80
Low	65	29(44.6)	1.12(0.67,1.85)	0.67
Tumor histological type	210	84(40.0)	1(Reference)	
Papillary	157	66(42.0)	1.05(0.72,1.54)	0.80
Nonpapillary	53	18(34.0)	0.85(0.47,1.53)	0.59

		TT		
		Cases (n=210)	n (%)	OR (95% CI)	*p*
Tumor stage	210	58(27.6)	1(Reference)	
Invasive	134	48(35.8)	1.30(0.84,2.01)	0.25
Superficial	76	10(13.2)	0.48(0.23,0.98)	0.04
Tumor grade	210	58(27.6)	1(Reference)	
High	145	42(29.0)	1.05(0.67,1.65)	0.84
Low	65	16(24.6)	0.89(0.48,1.66)	0.72
Tumor histological type	210	58(27.6)	1(Reference)	
Papillary	157	42(26.8)	0.97(0.62,1.52)	0.89
Nonpapillary	53	16(30.2)	1.09(0.58,2.05)	0.78

OR: odds ratio; CI: confidence interval.
